# Pulmonary blastoma in a pregnant woman: a case report and brief review of the literature

**DOI:** 10.1186/s12890-021-01804-z

**Published:** 2022-01-04

**Authors:** Michał P. Budzik, Grzegorz Panek, Małgorzata Bińkowska, Beata Osuch, Ewa Borkowska, Anna M. Badowska-Kozakiewicz

**Affiliations:** 1 Department of Gynaecologic Oncology and Obstetrics, Centre of Postgraduate Medical Education, 231 Czerniakowska, 00-416 Warsaw, Poland; 2 Department of Cancer Prevention, Medical University of Warsaw, 81 Żwirki i Wigury, 02-091 Warsaw, Poland; 3 Student Scientific Association, Medical University of Warsaw, 1 Oczki, 02-001 Warsaw, Poland

**Keywords:** Pulmonary blastoma, Histopathology, Case report, Pregnancy, Lung cancer, Haemoptysis

## Abstract

**Background:**

Pulmonary blastoma (PB) comprises a rare heterogeneous group of lung tumours typically containing immature epithelial and mesenchymal structures that imitate the embryonic lung tissue and extremely rarely occurs during pregnancy. Although cough and haemoptysis are the most common PB symptoms, they usually indicate other serious pregnancy-related complications.

**Case presentation:**

The article presents the unusual case of a 22-year-old pregnant woman diagnosed with PB during pregnancy.

**Conclusions:**

PB is characterized by poor prognosis and patients’ outcome relies on a rapid diagnosis. Surgery remains the most common and effective treatment. Due to the extreme rarity, the literature contains only single mentions of PB in pregnancy, thus its impact on the course of pregnancy and the developing fetus remains unknown.

## Background

The symptom of haemoptysis usually indicates a serious pathology and can occur with lung cancer, infections, vasculitis or certain cardiovascular conditions. When occurs in pregnancy, may be assumed to be caused by a pulmonary embolism, which remains the most common haemoptysis cause in pregnant women and is associated with maternal obstetric complications and high fetal risk resulting from intrauterine hypoxia and preterm birth. Although rare, lung tumors should not be overlooked in the presence of haemoptysis during pregnancy, especially considering the rare subtypes of cancer occurring in young women without the usual risk factors for lung cancer.

## Case presentation

**Fig. 1:**
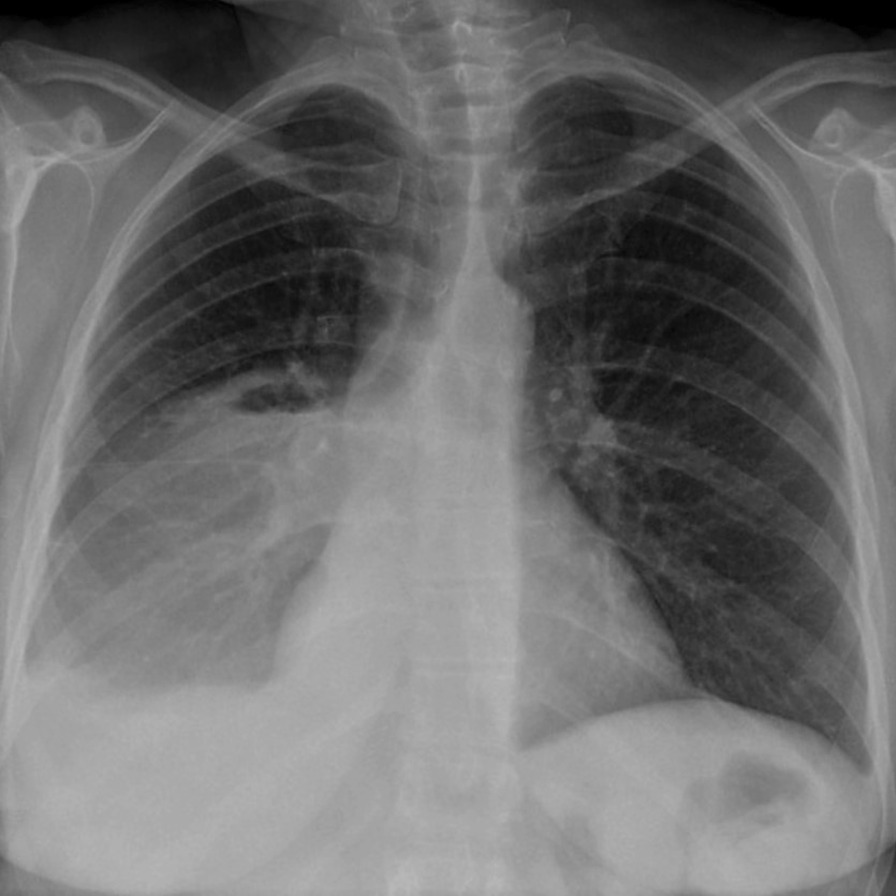
Chest X-ray showing the presence of solid mass peripherally in the lower lobe of the right lung

A 22-year-old woman (gravida 1) was referred to our hospital at 37 weeks’ gestation with chest pain, cough and haemoptysis. She had the medical history of SARS-CoV-2 asymptomatic infection underwent 3 weeks before. Currently, she was with no reported complications related to pregnancy. Her previous medical and family history was unremarkable, and she was a non-smoker. On physical examination, her breath sounds were diminished in the right lung zone. Vital signs were BP 112/68 mm Hg, heart rate 84 beats/min, respiratory rate 22 breaths/min, oxygen saturation 97% on room air and body temperature 36.4 °C. Arterial blood gases were within the normal range for pregnancy, while pCO2 remained slightly lowered compared to non-pregnant conditions (pO2 91.8 mmHg [reference range 65.0–99.0], pCO2 34.4 mmHg [35.0–45.0]). Pregnancy is a state of physiological respiratory alkalosis and compensatory metabolic acidosis. The resulting acid–base disturbances are compensated by the kidneys. Low levels of pCO2 in the maternal arterial blood facilitate the uptake of oxygen by the placenta. It is therefore an adaptive change that ensures the optimal concentration of oxygen in the fetal circulation [1]. Complete blood count and complete metabolic panel were normal (e.g. WBC 9.42 10^9^/L [4.0–10.0]; CRP 5.03 mg/l [0.00–10.00]; procalcitonin 0.02 ng/ml [< 0.5 ng/ml—low risk of severe sepsis and/or septic shock; > 2.0 ng/ml—high risk of severe sepsis and/or septic shock]. The PCR test for SARS-CoV-2 mRNA, QuantiFERON-Tuberculosis test and sputum culture were negative. At the presentation to the hospital, due to the patient's stable condition and obtained blood test results, pulmonary embolism and an infectious substrate were excluded. The chest X-rays showed the presence of a solid mass 9 cm in diameter peripherally in the lower lobe of the right lung with a slight displacement of the mediastinum to the left side and moderate pleural effusion (Fig. 1). Due to the increasing haemoptysis and development of a partial respiratory insufficiency (pO2 57.5 mmHg [65.0–99.0], pCO2 39.8 mmHg [35.0–45.0]), an urgent caesarean section was performed at 38 pregnancy week and a son in good condition was born (10 points on the Apgar score). The patient’s condition started to deteriorate due to increased bleeding and development of hypercapnic respiratory failure (pO2 53.1 mmHg [65.0–99.0], pCO2 48.3 mmHg [35.0–45.0]) so she was transferred to the thoracic surgery department. Contrast-enhanced chest computed tomography (CT) confirmed a 9.5 × 8.0 cm mass in the right lower lobe. The boundary of the lesion was clear and smooth, the enhancement was heterogenous, with evidence numerous necrotic foci. The mass abutted the pleura with no sign of invasion. There was no evidence of lymphadenopathy or metastases. A slight pleural effusion was described. The tumor was classified as clinical T4N0M0, stage IIIA according to the TNM classification of the Union of International Cancer Control (UICC), 8th edition.

An emergency right-side posterolateral thoracotomy with right lower lobectomy and lymph node dissection was done on the second day after caesarean section.

**Fig. 2:**
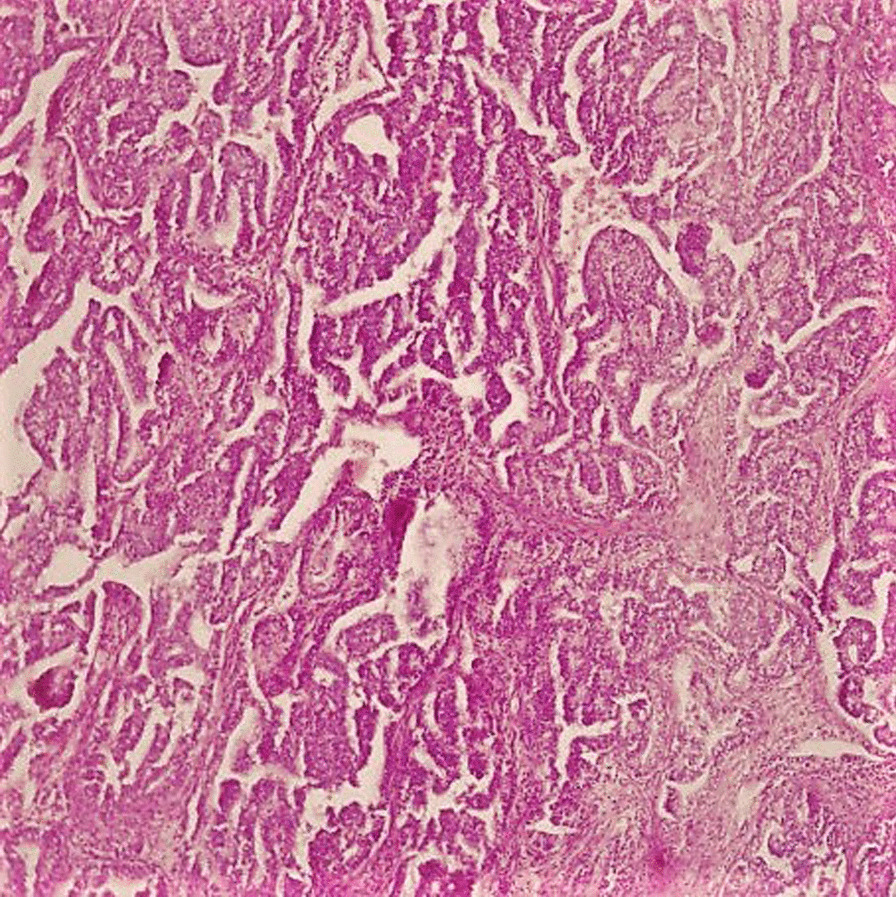
A biphasic pulmonary blastoma composed of malignant epithelial and mesenchymal component (H&E, 100× magnification)

**Fig. 3:**
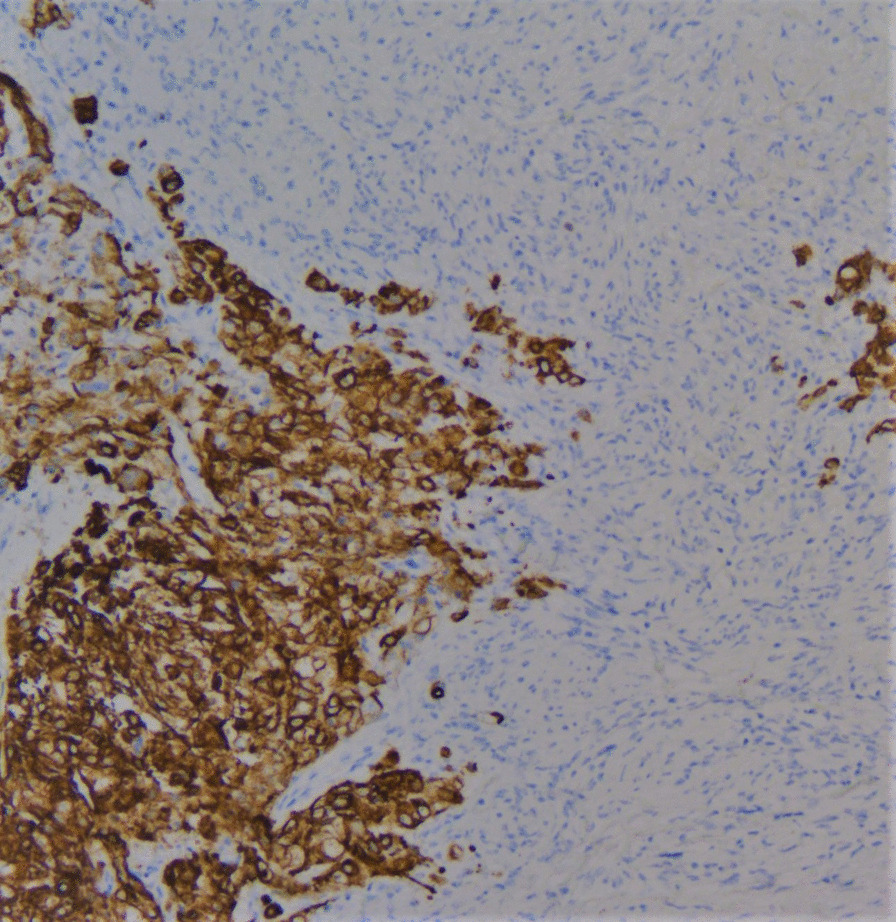
Immunohistochemical (IHC) analysis of CK 5/6 expression, 200× magnification

**Fig. 4:**
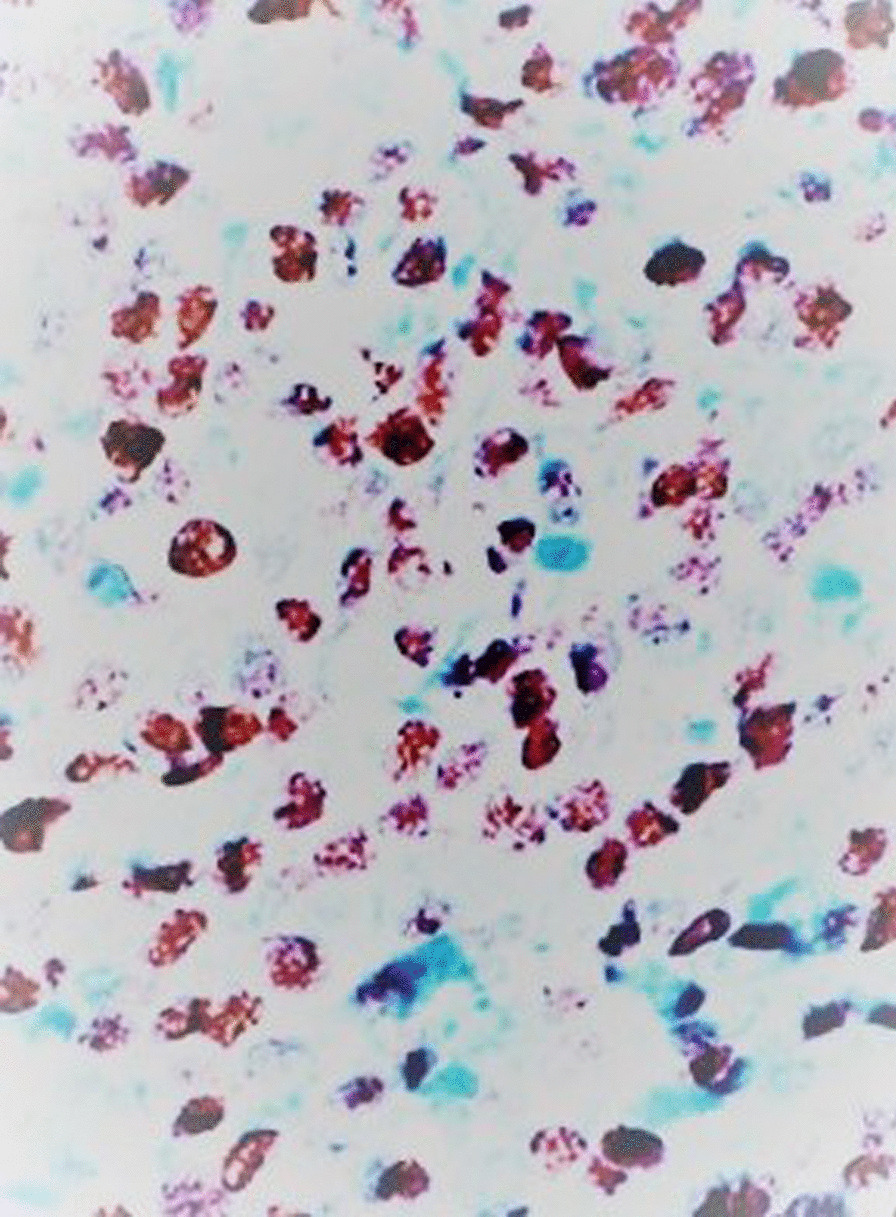
Immunohistochemical (IHC) analysis of myogenin expression, 400× magnification

**Fig. 5:**
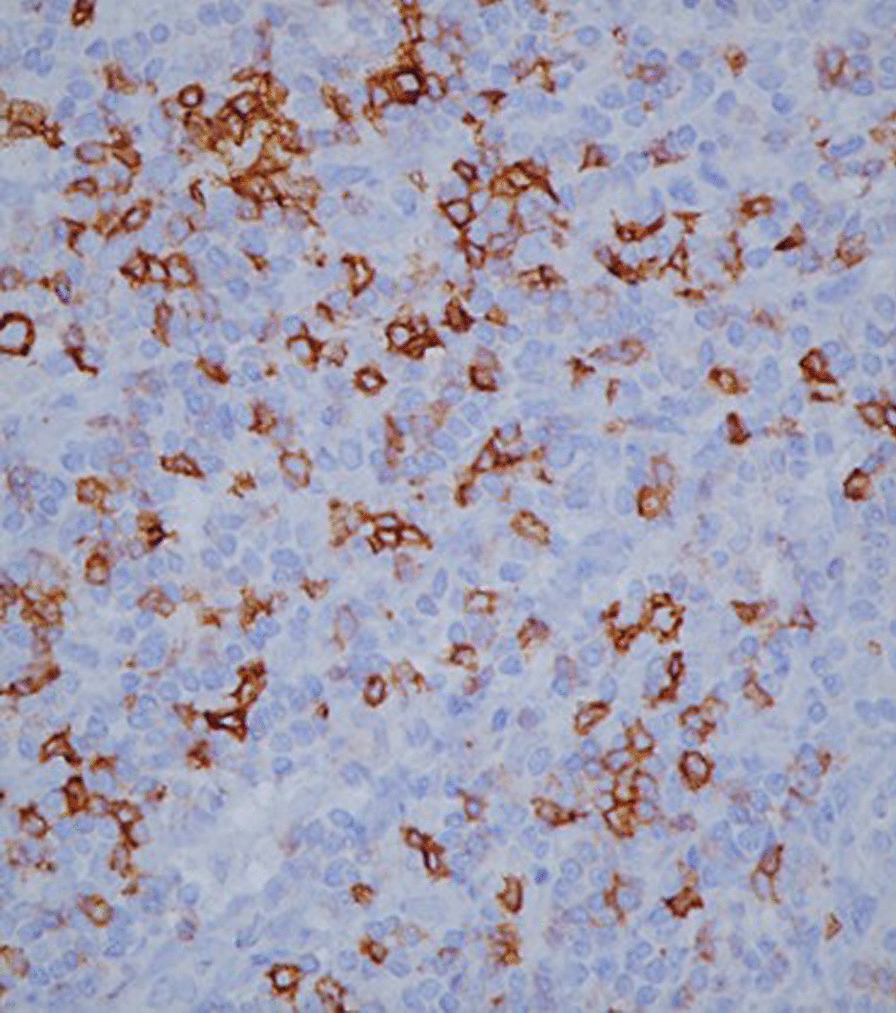
Immunohistochemical (IHC) analysis of PD-L1 expression, 400× magnification

On gross examination the tumor was well circumscribed unencapsulated mass, measured 10 × 8 cm. The cut surface of the lesion was heterogeneously white–grey with necrotic and multiple foci of hemorrhagic degeneration. Definitive histology with hematoxylin and eosin (H&E) staining demonstrated typical biphasic tumor with mixed epithelial and mesenchymal components (Fig. 2). The epithelial component formed glands/tubules with clear cytoplasm, whereas the mesenchymal component consisted of loose undifferentiated mesenchymal stroma with variable cellular atypia and foci of cartilage differentiation. Immunohistochemical (IHC) staining showed CK-5/6 (+) (Fig. 3), CK-7 (+), CD56 and vimentin (stromal cells), desmin (+), CD34 and CD99 (−), synaptophysin (−), myogenin (+) (Fig. 4) and PD-L1 (+) (Fig. 5). High mitotic activity was demonstrated in both epithelial and mesenchymal elements by using Ki-67 (up to 70%). The surgical margins and lymph nodes were negative.

Postoperative chest radiographs showed optimal lung expansion. Additional imaging studies did not reveal the presence of distant metastases. Subsequent histopathological examination confirmed a pulmonary blastoma with malignant mesenchymal stroma showing slight foci of a rhabdomyosarcomatous differentiation pTNM stage IIIb (T4, N0, M0). The patient was qualified for six courses of adjuvant chemotherapy with vincristine, actinomycin-D, and cyclophosphamide. At the end of planned number of chemotherapy courses and 6 months after surgery the patient remained clinically and radiologically asymptomatic.

## Discussion

Pulmonary blastoma (PB) comprises a rare heterogeneous group of lung tumours typically containing immature epithelial and mesenchymal structures that imitate the embryonic lung tissue resembling fetal lung of gestational age 10–15 weeks. PB accounts for 0.25–0.5% of all primary pulmonary malignancies. Based on the predominant tissue components, PB is divided into three subtypes:

- Well-differentiated fetal adenocarcinoma (WDFA), known as the monophasic pulmonary blastoma, consisting of epithelial component exclusively.
- Classic biphasic pulmonary blastoma (CBPB), comprising both epithelial and mesenchymal components, which is the most common subtype and currently considered as part of the spectrum of sarcomatoid carcinomas.
- Pleuropulmonary blastoma (PPB), characterized by the presence of mesenchymal components only [2].

Historically, the term pulmonary blastoma (PB) had included both pure fetal adenocarcinomas, pleuropulmonary blastomas as well as the classic biphasic blastomas. However recent WHO re-classifications separated well-differentiated fetal adenocarcinomas (WDFA) and pleuropulmonary blastomas (PPB) from the biphasic tumours (CBPB). Given the small number of cases and recent classification changes, interpreting the published clinical features and epidemiology of PB is challenging [3].

PB consists of an epithelial and mesenchymal stroma, where foci of chondrosarcoma, rabdomiosarcoma, osteosarcoma and yolk sac might also be found. Molecular studies indicate that epithelial and mesenchymal components may derive from a single precursor cell. Mutations in various genes (e.g., *TP53*, *EGFR*, *CTNNB1*) may be identified in some pulmonary blastomas [4]. Tumour specimens usually comprise areas of haemorrhage and necrosis, although translucent cytoplasm, tubular glandular cells and hyponuclear vacuoles might be demonstrated. The epithelial component of PB is composed mainly of tubules formed by non-ciliated glycogen-rich cells that mimic the pseudo-glandular stage of fetal lung development and typically does not express cytokeratins [5]. Different immunohistochemical staining methods in PB were studied by Larsen et al. The highest stain sensitivity was found with muscle actin (92%), vimentin (90%), neuron-specific enolase (NSE—83%), α-fetoprotein (AFP—82%), carcinoembryonic antigen (CEA—77%) and epithelial membrane antigen (EMA—71%) [6].

The aetiology of PB remains unknown, however over 80% of cases are associated with a history of smoking. Abnormalities in laboratory tests are usually non-specific and rare. No specific tumour markers for PB have been found yet. Some reports presented elevated AFP and CEA serum levels [7].

The most frequent PB symptoms include cough, haemoptysis, chest pain, dyspnoea and fever of unknown origin, although 40% of cases remain asymptomatic and are revealed during incidental chest X-rays. In the vast majority of cases, the tumour is one-sided, with a higher prevalence in superior lobes. The mean tumour diameter at the time of primary diagnosis is 7–10 cm. Typically radiological images show well-circumferenced mass displacing the mediastinum while CT-scans reveal dense and vesical elements with varying contrast uptake. Endobronchial growth is present in approximately ¼ of cases and even rarely pleura invasion is present. Differential diagnosis should include benign lesions such as pleural fibroma or hamartoma as well as other malignancies (primary lung cancers, metastases) [3, 8]. PB symptoms may occur at any age, but 80% of cases are diagnosed in adults, usually in the fourth decade of life and shows a strong female predominance, which is thought to be caused by the influence of estrogen through its receptors overactivated by β-catenin [9].

Diagnosis is made with transbronchial biopsy but obtaining the representative tissue sample is possible only in approximately 25% of cases because of the PB peripheral nature. Surgery is the preferred method of treatment as pulmonary blastoma typically is a well-demarcated peripheral mass. The range of surgery should be determined individually and depends on the tumour size, pleural invasion, lymph node metastasis and comorbidities. The average survival rate among operated patients is 33 months, as compared to 2 months’ survival in non-operated patients. Limited lobectomies are associated with better survival rates than pneumonectomies, probably due to primary reduced tumour burden [10].

Larsen et al. reported a 16% response rate to chemotherapy in PB. So far, neither of the agents is more effective than another, although cisplatin is found in most treatment regimens, as it has been proved to improve prognosis in other germ cell tumours [6]. The selection of the most effective chemotherapeutics remains a major problem as the PB often presents biphasic structure with both epithelial and mesenchymal components, sensitive to different lines of treatment [11].

Overall PB prognosis is poor, the 2-year survival rate is 34%, and the 5-year survival rate is 16%, respectively. Overall prognosis depends primarily on the tumour size and distant organs involvement. Biphasic type of tumour (CBPB), metastatic disease at the time of diagnosis, tumour size exceeding 5 cm, early relapse (within 12 months after treatment) and lymph node involvement contribute to the unfavourable prognosis. At the time of diagnosis distant metastasis is present in 43% of patients and mainly concerns the brain, pleura, mediastinum, diaphragm and liver [12, 13].

Cancer in pregnancy is an increasingly common phenomenon faced by oncologist. This is a consequence of postponed motherhood until a later age and high rates of malignant tumors in the group of young women. Recent studies revealed the lack of knowledge among medical personnel and concerns about possible fetal damage caused by diagnostic radiology. Due to the possibilities of modern diagnostic equipment, also cancer radiological diagnosis in the first trimester of pregnancy is not contraindicated. According to Pereg et al. [14], performing radiological diagnostic procedures involving fetal exposure to ionizing radiation doses lower than 0.1 or even 0.2 Gy (10–20 cGy) does not increase the risk of congenital defects. The risk of birth defects decreases with increasing gestational age. Minimizing the effects caused by ionizing radiation by further reducing the dose and the residence time, increasing patient’s distance from the radiation source as well as using a thicker shield and a less active radiation source allow the use of radiological imaging methods regardless of the stage of pregnancy [14, 15].

## Conclusions

Pulmonary blastoma is an extremely rare malignant neoplasm characterized by poor prognosis. PB grows rapidly and patients’ outcome relies on a rapid diagnosis what brings serious difficulties because of the rarity of the disease. Surgery remains the most common and effective treatment. Due to the extreme rarity, the literature contains only single mentions of PB in pregnancy, thus its impact on the course of pregnancy and the developing fetus remains unknown.

The presented case report is unique because it presents a very rare case of PB diagnosed during pregnancy, although it confirms that lung tumors should be included in the differential diagnosis of such patients, obviously after excluding other complications typical for the pregnancy.

The primary “take-away” lessons of this case report:

- Pregnancy is a state of physiological respiratory alkalosis and compensatory metabolic acidosis. The resulting acid–base disturbances are compensated by the kidneys. Low levels of pCO2 in the maternal arterial blood facilitate the uptake of oxygen by the placenta. It is therefore an adaptive change that ensures the optimal concentration of oxygen in the fetal circulation.
- Despite its very rare occurrence, the possibility of lung cancer in young pregnant women should not be forgotten.
- Haemoptysis in pregnant women, regardless of the stage of pregnancy, may be associated with the presence of a lung tumour.
- In the third trimester, the optimal management of pulmonary blastoma consists of delivery by caesarean section, stopping lactation and implementation of surgery treatment.

## Data Availability

All data generated or analysed during this study are included in this published article.

## Abbreviations

PB: Pulmonary blastoma
WDFA: Well-differentiated fetal adenocarcinoma
CBPB: Classic biphasic pulmonary blastoma
PPB: Pleuropulmonary blastoma
